# Editorial: Socio-emotional skills in relation to aggressive and prosocial behaviors: From early childhood to adolescence

**DOI:** 10.3389/fpsyg.2022.1055948

**Published:** 2022-10-13

**Authors:** Carmen Belacchi, Paola Molina, Nicoletta Businaro, Eleonora Farina

**Affiliations:** ^1^Department of Communication Sciences, Humanities and International Studies, University of Urbino Carlo Bo, Urbino, Italy; ^2^Interuniversity Department of Regional and Urban Studies and Planning, University of Turin, Turin, Italy; ^3^Faculty of Social Studies, Vitenskapelig, Internasjonal og Diakonal Specialized University, Oslo, Norway; ^4^Department of Human Sciences for Education “Riccardo Massa”, University of Milano Bicocca, Milan, Italy

**Keywords:** socio-emotional skills, aggressive behavior, prosocial behavior, early childhood, adolescence

Two theoretical assumptions motivate the choice of the present Research Topic focused on the role of “socio-emotional” skills in the development of interpersonal relationships from preschool to adolescence:

1. The assumption that the emotional dimension plays a key role in the psychological development of a person;
2. The importance of peers' interactions in the structuring/expression of socio-cognitive and self-regulatory skills, essentials for the individual and collective wellbeing.

In the following, we reflect on possible explanations of the mentioned assumptions.

1. In the field of developmental psychology, the attention on socio-emotional skills has been addressed relatively late compared to other psychological abilities, such as, specifically, cognitive abilities. In fact, the development of intelligence has traditionally been considered more qualifying and distinctive for the human experience.
  Moreover, psychological science focused on the study of general competencies, like cognitive and linguistic skills, that are considered fundamental for the development of more specific competencies (see Whorf, 1956).
  Since the 1970s, the emotional skills are considered a remarkable trigger for psychological processes. Empirical evidence supports the assumption that emotional development can constitute a potential risk factor and/or a protective factor for adaptive behavior, and individual and social wellbeing. The acknowledgment of the importance and the specificity of the emotional skills has contributed to redefining the relationship between emotion and intelligence, through the new theoretical constructs of emotional intelligence (Salovey and Mayer, 1990; Goleman, 1995) and cognitive empathy (Hogan, 1969; Saarni, 1999). The new integrated approach, which highlights both the complexity and the peculiarities of a person, has brought theoretical and practical implications for: the prevention of psychological/behavioral disorders and for clinical interventions especially in the early stages of psychological development when personality is still under construction.
2. Emotional skills, such as the ability to understand and express emotions and affections, are intrinsically and inextricably associated with experiences in the family, especially with the maternal figure. The studies regarding the interactions among peers had initially focused on the aggressive behavior related to this kind of relationship. Nowadays, empirical evidence points out the relevant role of peers' interactions in the development of emotions' understanding and expression, the self-regulatory capacity, and the ability to recognize own and others' needs (Dunn, 1988). The comparison with peers supports the perspective-taking ability, the self-agency, and self-identity processes, and can lead both into an aggressive-competitive direction and into the form of altruism and prosocial behavior. Recently, studies about bullying have acquired great prominence in the field of developmental psychology and have contributed to the identification of specific roles in group interactions, such as aggressive-hostile and altruistic-prosocial roles. These interpersonal behaviors are interrelated with both socio-contextual and psycho-individual factors, in particular with the perspective-taking ability and affective empathy.

The present special issue provides an overview of the most recent research on the socio-emotional skills for social adaptation and psycho-social wellbeing during childhood and adolescence; considering that these skills are crucial for the development of a sense of identity and for achieving adequate personal and social adjustments.

This special issue presents 13 studies covering a wide range of ages, from preschool (*N* = 5 studies), primary school (*N* = 1), to adolescence (*N* = 4) and late adolescence (*N* = 2); 1 study analyses the entire age span from preschool (5 years) to the late adolescence (17 years).

Eleven papers present original empirical research and two contributions are systematic reviews.

In their study with Italian adolescents, Giancola et al. investigate the mediating effect of trait emotional intelligence (TEI) on the association between the dark triad (Machiavellianism, psychopathy, and narcissism) and prosocial sustainability, declined in terms of altruism and equity. Main findings suggest that TEI might reduce the malevolent effects of the dark triad on altruism and equitable behavior in late adolescence.

Salerni and Caprin present research that enrolled 160 Italian pre-schoolers and their teachers. The study investigates whether the early day-care experience can influence the prosocial behaviors that children exhibit during free-play social interactions with peers; and the associations between the enactment of prosocial behaviors and social-emotional and behavioral competence. The findings are interesting in light of the possible associations between socialization outside of the family context, prosocial behavior, and children's socio-emotional skills. Di Norcia et al. examine the representations on friendship among Italian children aged 6–11 years by depicting themselves with a close friend in two relational situations: wellbeing and distress. Some indices of children's drawings were predictive of their tendency to enact physical and verbal aggression: for instance, the capacity to relate with one's own friend even in difficult times predicts lesser aggression with peers.

Farina and Belacchi explore the longitudinal effects of interpersonal variables (social status indices) and personal variables (empathy and understanding of emotions) on role-taking in bullying episodes (hostile, prosocial, victim, and outsider roles) in the transition between kindergarten and primary school. The study highlights the existence of independent effects of two social status indices on the participant role-taking in bullying episodes.

An intervention study aimed to promote perspective-taking ability in Italian children who are victims of psychological maltreatment, is presented in Cigala and Mori's paper. The study contributes to the limited and controversial research on this topic. The perspective-taking represents a significant protective factor that improves the social adaptation of preschool children who are victims of psychological abuse.

Grazzani et al. investigate the role of socio-emotional skills and resilience in explaining mental health in 778 Italian adolescents, during the COVID-19 pandemic. The paper fits in the field of studies which contribute to explore the consequences of the pandemic and point out that resilience may be an important protective factor against the incidence of internalized disorders in the face of adversity.

Simon and Nader-Grosbois in their explorative study, carried out in Belgium, examine parents' representations of their preschool child's empathy, personality, and social (mal)adjustment. Main findings reveal no significant differences between mothers' and fathers' representations of their child, but significant gender differences emerge, with girls being more skilled in affective empathy and boys in cognitive empathy. In general, empathy emerges to be positively related to social adjustment.

Shin examines the additive and interactive effects of 677 early adolescents' social achievement goals and perceived relational support from teachers and peers on their social behavior, in South Korea. Findings indicate that individual differences in psychological processes of social goals and perceived relatedness matter for youth's social adjustment. The findings also emphasize the need to consider adolescents' social goals in conjunction with their perceptions of the relational features of their interpersonal environments.

Fernández-Martín et al. contribute with their work to synthesize research on the efficacy and effectiveness of socio-emotional skills programs in Ibero-American contexts in early childhood. The systematic review of 22 empirical studies shows that the social and emotional learning (SEL) variables with the highest incidence and significant results are: self-awareness; social awareness; self-control; relationship skills; decision-making; school climate; wellbeing; and academic achievement. The study also identifies some factors that can ensure the success of future SEL programs.

Bølstad et al. present a pilot study on the first experimentation in the Scandinavian population of an emotion-focused intervention “Tuning in to Kids (TIK)” addressed to parents. Their findings suggest an effect on the improvement in parents' emotion coaching and their appraisal of child externalizing problems, while children's self-regulation showed mainly normative developmental improvements.

Wang et al. investigate the relationship between emotional intelligence and prosocial behavior among Chinese adolescents, examining the mediating effect of social support and the moderating effect of self-esteem in this relationship. They found a positive association between emotional intelligence and prosocial behavior; furthermore, the more social support college students receive, the more they tend to engage in prosocial behavior, especially in students with high self-esteem.

Deng et al. conducted a meta-analysis on the association between empathy and defending behavior in adolescent bullying. Main results highlight a significant correlation between the two variables, whose strength was moderated by the kind of empathy (affective empathy has a stronger association than cognitive) and by the evaluator of defending behavior (stronger association when defending was self-evaluated).

Xiong et al. explore the association between the sense of deprivation and prosocial tendencies in a large sample of 1,630 Chinese children who migrate from rural to urban areas and may experience social exclusion, prejudice, and discrimination. The study offers evidence that can be relevant for parents, educators, and other members of the society who are concerned about migrant children's psychosocial adaptation.

In conclusion, this Research Topic is intended to provide a broad and articulated overview of current research on the role of emotional-social skills in altruistic and/or hostile behaviors among peers. We realize that there is a heterogeneity of both the age groups considered (from preschool to adolescence) and the aspects investigated (emotional intelligence, empathy, adjustment, resilience, parent educational training, social status, perceived social support and group identity, social and emotional learning, early socialization experience). Therefore, we would like to encourage researchers to carry out systematic and specific reviews on studies over the various aspects investigated in this Research Topic.

## Author contributions

All authors equally contributed to the conception and the writing of this editorial. All authors contributed to the article and approved the submitted version.

## Conflict of interest

The authors declare that the research was conducted in the absence of any commercial or financial relationships that could be construed as a potential conflict of interest.

## Publisher's note

All claims expressed in this article are solely those of the authors and do not necessarily represent those of their affiliated organizations, or those of the publisher, the editors and the reviewers. Any product that may be evaluated in this article, or claim that may be made by its manufacturer, is not guaranteed or endorsed by the publisher.

## References

[B1] Dunn J. (1988). The Beginnings of Social Understanding. Harvard University Press.

[B2] Goleman D. (1995). Emotional Intelligence. New York, NY: Bantam Books.

[B3] Hogan R. (1969). Development of an empathy scale. J. Consult. Clin. Psychol. 33, 307. 10.1037/h0027580 4389335

[B4] Saarni C. (1999). The Development of Emotional Competence. New York, NY: Guilford press.

[B5] Salovey P. Mayer J. D. (1990). Emotional intelligence. Imaginat. Cognit. Person. 9, 185–211. 10.2190/DUGG-P24E-52WK-6CDG

[B6] Whorf B. L. (1956). Language, Thought, and Reality: Selected Writings of …. (Edited by John B. Carroll.).18316728

